# Two-Stage Marker Detection–Localization Network for Bridge-Erecting Machine Hoisting Alignment

**DOI:** 10.3390/s25175604

**Published:** 2025-09-08

**Authors:** Lei Li, Zelong Xiao, Taiyang Hu

**Affiliations:** School of Electronic and Optical Engineering, Nanjing University of Science and Technology, Nanjing 210094, China

**Keywords:** bridge-erecting machine alignment, two-stage detection–localization, Transformer homography estimation

## Abstract

To tackle the challenges of complex construction environment interference (e.g., lighting variations, occlusion, and marker contamination) and the demand for high-precision alignment during the hoisting process of bridge-erecting machines, this paper presents a two-stage marker detection–localization network tailored to hoisting alignment. The proposed network adopts a “coarse detection–fine estimation” phased framework; the first stage employs a lightweight detection module, which integrates a dynamic hybrid backbone (DHB) and dynamic switching mechanism to efficiently filter background noise and generate coarse localization boxes of marker regions. Specifically, the DHB dynamically switches between convolutional and Transformer branches to handle features of varying complexity (using depthwise separable convolutions from MobileNetV3 for low-level geometric features and lightweight Transformer blocks for high-level semantic features). The second stage constructs a Transformer-based homography estimation module, which leverages multi-head self-attention to capture long-range dependencies between marker keypoints and the scene context. By integrating enhanced multi-scale feature interaction and position encoding (combining the absolute position and marker geometric priors), this module achieves the end-to-end learning of precise homography matrices between markers and hoisting equipment from the coarse localization boxes. To address data scarcity in construction scenes, a multi-dimensional data augmentation strategy is developed, including random homography transformation (simulating viewpoint changes), photometric augmentation (adjusting brightness, saturation, and contrast), and background blending with bounding box extraction. Experiments on a real bridge-erecting machine dataset demonstrate that the network achieves detection accuracy (mAP) of 97.8%, a homography estimation reprojection error of less than 1.2 mm, and a processing frame rate of 32 FPS. Compared with traditional single-stage CNN-based methods, it significantly improves the alignment precision and robustness in complex environments, offering reliable technical support for the precise control of automated hoisting in bridge-erecting machines.

## 1. Introduction

In modern bridge engineering, bridge-erecting machines serve as core equipment for the hoisting of precast girders/bridge panels, where the alignment during hoisting directly impacts the construction efficiency and operational safety. In automated construction scenarios, the high-precision detection and localization of markers (e.g., positioning targets, reference lines) is critical in achieving accurate docking between bridge-erecting machines and precast components. However, the complexity of real construction environments poses significant challenges for visual perception systems: dynamic lighting conditions (e.g., strong light reflection, shadow occlusion), random occlusion by dust/mechanical parts, and the surface contamination of markers after long-term use can all lead to false detection, missed detection, or localization errors in traditional vision methods.

Additionally, the dual requirements of real-time performance (to match the equipment movement speed) and alignment precision (millimeter-level error tolerance) make it difficult for traditional single-stage detection–localization methods based on convolutional neural networks (CNNs) to balance efficiency and robustness. Furthermore, the scarcity of annotated data in real construction scenarios (limited by construction cycles, safety regulations, etc.) further restricts the generalization capabilities of models.

To address the high precision requirements and data scarcity in marker detection–localization under complex construction environments, this paper proposes a two-stage marker detection–localization network for bridge-erecting machine hoisting alignment, with three key innovations. First, a “coarse detection–fine estimation” phased architecture is designed: the first stage uses a lightweight detection module with a dynamic hybrid backbone and adaptive feature switching to filter background interference and generate coarse marker region boxes; the second stage constructs a Transformer-based homography estimation module, which leverages self-attention to capture long-range dependencies between marker keypoints and the scene context, enhancing multi-scale feature interaction and positional encoding to achieve the end-to-end learning of precise homography matrices. Second, a multi-dimensional data augmentation strategy simulates lighting distortion, occlusion, and marker contamination to generate diverse training samples, improving the model’s adaptability to complex environments.

Experimental results show that the proposed method achieves detection accuracy (mAP) of 97.8%, a reprojection error of less than 1.2 mm in homography estimation, and a processing frame rate of 32 FPS on a real bridge-erecting machine construction dataset. Compared with traditional single-stage methods, it significantly enhances the alignment precision and robustness in complex environments, providing reliable technical support for the precise control of automated hoisting in bridge-erecting machines.

## 2. Related Works

Marker detection and localization under complex construction environments have been a long-standing challenge in computer vision and construction robotics. This section reviews key advancements in three interrelated areas, namely traditional feature-based methods, deep learning-driven detection–localization frameworks, and data augmentation strategies, while highlighting their limitations in addressing the specific demands of bridge-erecting machine hoisting alignment.

**Traditional Feature-Based Methods.** Early efforts in marker detection relied on handcrafted feature extraction and geometric matching. The Scale-Invariant Feature Transform (SIFT) [1], a classic local feature descriptor, has been widely used to detect and match keypoints for homography estimation. SIFT’s invariance to scale and rotation makes it robust to viewpoint changes, but it struggles with dynamic lighting variations (e.g., strong reflections or shadows) and partial occlusion—common in construction sites—due to its sensitivity to intensity changes. Similarly, Harris corner detectors [2] focus on local edge responses for corner detection, but they fail to capture the global context, leading to unstable performance when markers are contaminated (e.g., by dust or paint peeling). Other feature point extractors include Speeded-Up Robust Features (SURF) [3], Oriented FAST and Rotated BRIEF (ORB) [4] and the Boosted Efficient Binary Local Image Descriptor (BEBLID) [5]. ORB builds on two foundational techniques: the Features from Accelerated Segment Test (FAST) method, a rapid corner detector that identifies keypoints by comparing the pixel intensities in a circular region, and Binary Robust Independent Elementary Features (BRIEF), a descriptor that generates binary feature vectors via random pixel pair comparisons. ORB enhances these with rotation invariance, making it suitable for real-time scenarios. These methods are widely used for marker detection but share similar limitations in complex construction environments.

**Deep Learning for Detection–Localization.** The rise of deep learning has spurred the development of end-to-end detection–localization frameworks. Single-stage detectors like You Only Look Once (YOLO) [6] and the Single-Shot MultiBox Detector (SSD) [7] achieve real-time speeds by directly predicting bounding boxes and class scores in a single pass. However, their focus on efficiency often sacrifices precision, especially for small or occluded markers. For instance, YOLOv8 [8], a state-of-the-art single-stage model, struggles to distinguish markers from cluttered backgrounds (e.g., machinery or debris) in construction scenes, resulting in high false-positive rates.

Two-stage frameworks, such as the Faster Region-Based Convolutional Neural Network (Faster R-CNN) [9], improve the accuracy by first generating region proposals and then refining them. Faster R-CNN’s region proposal network (RPN) better handles object scale variations, but its computational complexity limits real-time performance—critical in aligning fast-moving hoisting equipment. Moreover, both single- and two-stage CNN-based methods rely on convolutional layers, which inherently model local spatial correlations but fail to capture long-range dependencies between marker keypoints and the scene context (e.g., the relationship between marker corners and nearby machinery). This limitation restricts their ability to estimate precise homography matrices under occluded or contaminated conditions.

**Homography Estimation with Deep Learning.** Homography estimation, a core component of marker localization, has been explored using CNNs. Recent advances in deep learning have revolutionized homography estimation by replacing traditional multi-step pipelines with end-to-end trainable frameworks. These methods can be broadly categorized into supervised, unsupervised, and hybrid approaches (e.g., self-supervised, semi-supervised), each addressing distinct challenges in geometric alignment [10].

Supervised methods leverage synthetic or labeled datasets to train networks for direct homography regression. Early work by DeTone et al. [11] introduced the four-point parameterization of the homography matrix, enabling efficient network training by regressing offsets of keypoints rather than the full 3 × 3 matrix. Subsequent studies optimized network architectures for efficiency, such as lightweight models based on ShuffleNet [12], which reduced the parameters to under 9 MB while maintaining accuracy, making them suitable for edge devices. Hybrid frameworks like HomoNetComb [13] combined deep learning with energy minimization, using CNNs to predict initial homographies and gradient descent to refine residuals, balancing speed and precision. Transformer-based models further improved the performance by capturing long-range feature dependencies, with attention mechanisms enhancing alignment in large-baseline scenarios [14]. However, supervised methods face limitations in generalizing to real-world scenes due to the scarcity of labeled data and synthetic-to-real domain gaps.

Unsupervised methods eliminate the need for labeled data by optimizing the photometric consistency between warped and target images. A key innovation was the introduction of homography flow [15], a low-rank representation of optical flow constrained by the homography subspace, enabling robust estimation by focusing on dominant planar motion. Generative adversarial networks (GANs) were also applied to enforce coplanarity constraints, where discriminators distinguish between original and warped images to guide homography prediction [16]. Content-aware masks [17] and contextual correlation layers [18] were integrated to suppress dynamic objects and occlusions, improving the robustness in low-texture or noisy environments. Multi-scale and cascaded network structures [19] further refined coarse-to-fine estimation, reducing the reprojection errors for large displacements. Despite progress, unsupervised methods struggle with training stability and may fail in scenes with significant depth variations.

Despite advancements, existing deep learning methods face trade-offs between accuracy, speed, and robustness in dynamic construction environments. Supervised models lack adaptability to unlabeled real-world data, while unsupervised approaches struggle with large parallax and occlusion. Single-stage CNNs often sacrifice precision for efficiency, failing to meet the millimeter-level alignment requirements of bridge-erecting machines. Thus, a two-stage framework combining lightweight detection and Transformer-based fine estimation is proposed to address these challenges.

**Computer Vision in Civil Engineering and Construction.** Computer vision is widely applied in civil engineering and construction, facilitating tasks like safety monitoring (e.g., detecting non-hardhat wearers [20]), tracking workers and equipment [21], and progress monitoring via 3D point clouds [22]. It enables activity recognition for earthmoving operations [23] and interactions between excavators and dump trucks [24]. Techniques include CNNs for object detection [25] and two-stream networks for worker activity recognition [26], using data from cameras, scanners, and unmanned aerial vehicles (UAVs) [27,28]. These applications address site complexity, enhancing safety, productivity, and decision making.

**Data Augmentation for Construction Scenes.** Data scarcity is a critical bottleneck in training robust models for construction environments, where annotated images are limited due to safety regulations and long construction cycles. Synthetic data generation, such as the SYNTHIA dataset [29] for urban scenes, uses 3D rendering to simulate diverse environments. However, these datasets focus on urban settings (e.g., streets and buildings) and fail to simulate construction-specific interferences like dust occlusion, marker contamination, or dynamic lighting from heavy machinery. Existing augmentation strategies for object detection (e.g., random cropping or flipping) [30] also do not address construction-specific challenges, leaving models poorly adapted to real-world scenarios.

## 3. Methodology

### 3.1. Overall Architecture

**Figure 1:**
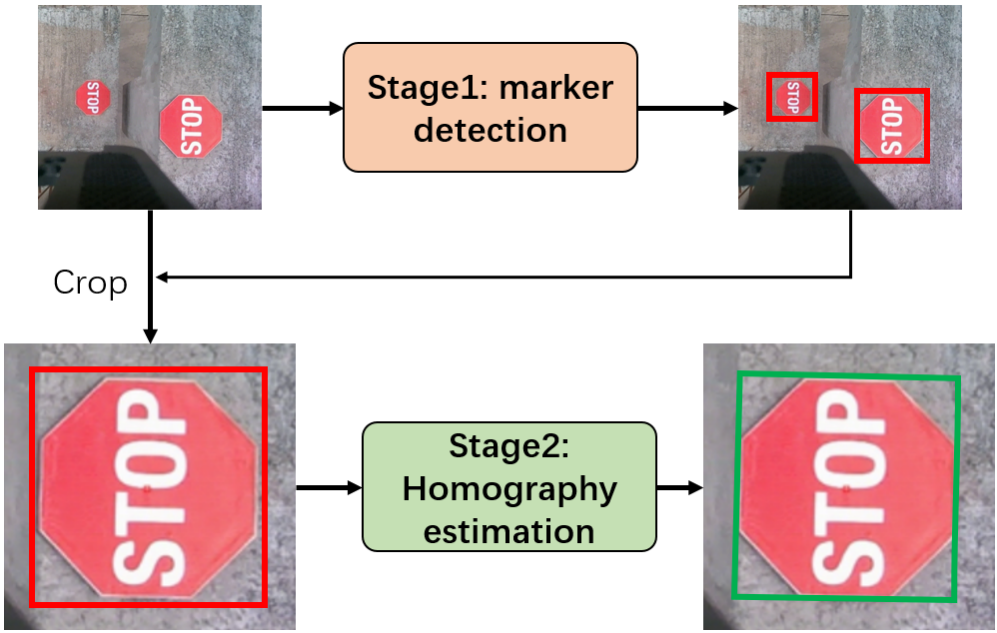
The overall framework of the proposed method. Red boxes denotes the results of marker detection, green boxes denotes the results of homography estimation.

The proposed framework adopts a two-stage “coarse detection–fine estimation” architecture (Figure 1).

- Stage 1: Lightweight Marker Detection Module for the rapid coarse localization of markers in complex environments.
- Stage 2: Transformer-Based Homography Estimation Module for precise coordinate transformation between markers and hoisting equipment.

### 3.2. Stage 1: Lightweight Marker Detection Module

**Figure 2:**
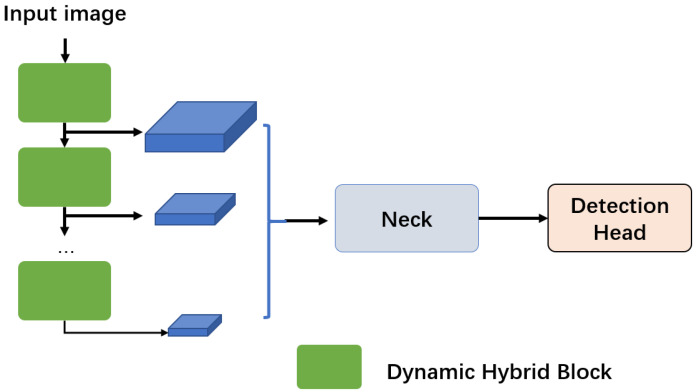
The structure of the marker detection network.

As shown in Figure 2, this stage focuses on efficient coarse localization through the dynamic hybrid block, which consists of two key components: a dynamic hybrid backbone (DHB) and a dynamic switching mechanism. Multi-scale feature maps are generated by the dynamic hybrid blocks, and these feature maps are further processed by the neck and detection head, which are consistent with YOLOv8 [8].

#### Dynamic Hybrid Block (DHB)

**Figure 3:**
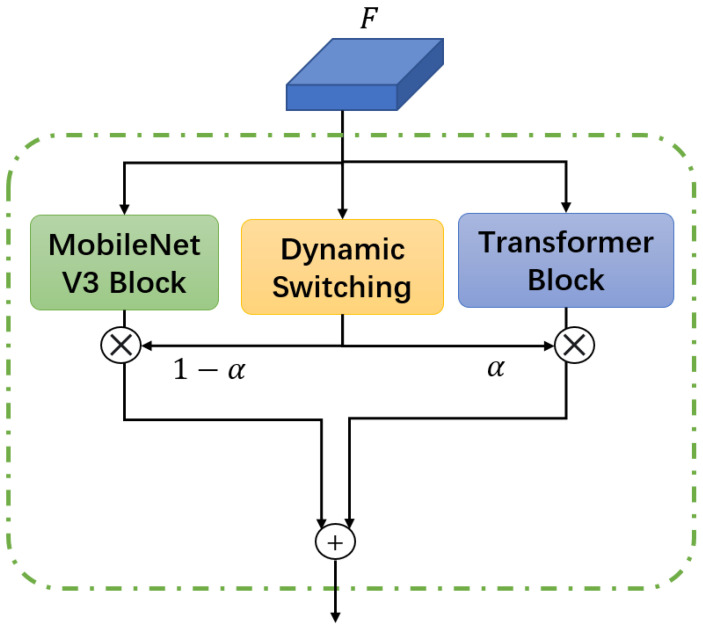
The detailed structure of the dynamic hybrid block.

To balance geometric feature extraction and complex texture modeling, the DHB dynamically switches between convolutional and Transformer branches based on feature complexity, as shown in Figure 3. The input feature is processed by two branches of feature processing network and a dynamic switching block. The two-branch network is designed as follows:

- The first branch uses MobileNetV3’s depthwise separable convolutions to extract geometric features (edges, corners). The depthwise convolution operation is defined as
  (1) $$F_{dw}=W_{dw}⊛X+b_{dw}$$
  where $W_{dw}\in R^{k\times k\times C}$ is the depthwise kernel, *k* is the kernel size, *C* is the input channel, ⊛ denotes depthwise convolution, and $b_{dw}$ is the bias.
- The second branch introduces lightweight Transformer blocks with scene-aware attention. The window multi-head self-attention (W-MSA) is computed as
  (2) $$W-\mathrm{MSA}\left(Z\right)=\mathrm{Concat}\left({\mathrm{head}}_{1},\ldots ,{\mathrm{head}}_{h}\right)W^{O}$$
  where ${\mathrm{head}}_{i}=\mathrm{Attention}\left(ZW_{i}^{Q},ZW_{i}^{K},ZW_{i}^{V}\right)$, and a scene prior bias *B* (encoding marker aspect ratio/position distribution) is added to the attention scores:
  (3) $$\mathrm{Attention}\left(Q,K,V\right)=\mathrm{Softmax}\left(\frac{QK^{⊤}}{\sqrt{d_{k}}}+B\right)V$$

For the dynamic switching module, a feature complexity discriminator calculates the entropy H(F) of feature map *F*:

(4) $$H\left(F\right)=-\sum_{i=1}^{H\times W}\left(\frac{F_{i}}{{∥F∥}_{1}}\log\frac{F_{i}}{{∥F∥}_{1}}\right)$$

The gating weight α∈[0,1] (via a sigmoid function) determines the branch contribution:

(5) $$\alpha =\sigma \left(\frac{H(F)-\tau }{\delta }\right),\;F_{\mathrm{stage}}=\alpha \cdot F_{\mathrm{trans}}+\left(1-\alpha \right)\cdot F_{\mathrm{conv}}$$

where τ=3.5 (threshold), δ=0.5 (scaling factor). The threshold τ acts as a criterion to judge the feature complexity. It determines whether the input feature maps have more high-level semantic information (when entropy H(F)>τ) or low-level geometric information (when H(F)<τ). The scaling factor (δ=0.5) controls the smoothness of weight switching via the sigmoid function. A smaller δ causes the weight change to be steeper. Here, it balances sensitivity and smoothness for stable adaptation to complex construction scenarios.

Threshold τ=3.5 is derived from an entropy distribution analysis of 500 training images, where H(F)>3.5 indicated high-level semantic features (e.g., cluttered backgrounds) and H(F)<3.5 indicated low-level geometric features (e.g., marker edges). Testing τ∈{2.5, 3.0, 3.5, 4.0} showed that this value minimized feature type misclassification.

Scaling factor δ=0.5 controls sigmoid smoothness. Evaluating δ∈{0.3, 0.5, 0.7} revealed that this value balanced sensitivity to feature changes and training stability, avoiding abrupt weight fluctuations in mixed-complexity scenarios (e.g., partially occluded markers).

### 3.3. Stage 2: Transformer-Based Homography Estimation Module

**Figure 4:**
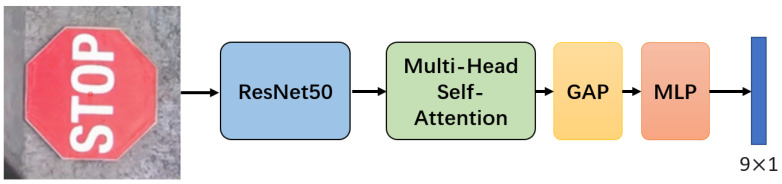
Framework of the Transformer-based homography estimation module. Input ROIs are processed by ResNet50 to extract multi-scale features, which are fed into a Transformer encoder with fused positional encoding. Global average pooling (GAP) and a multi-layer perceptron (MLP) then regress the 3 × 3 homography matrix.

This stage refines marker localization using the coarse detection results from Stage 1, focusing on modeling long-range dependencies between marker keypoints and the scene context—critical for precise homography estimation under occlusion or contamination. The module architecture (Figure 4) consists of three core components: input feature processing, a Transformer encoder with fused positional encoding, and a homography decoding head.

#### 3.3.1. Input Processing

Regions of interest (ROIs) cropped from the Stage 1 outputs (resized to 256 × 256) are fed into ResNet50’s convolutional layers (C3–C5), which extract multi-scale features with 256, 512, and 1024 channels, respectively. These features are concatenated and flattened into a sequence $X\in R^{H\times W\times C}$, where H=W=16 and C=1024 for the final feature map, preserving the spatial and semantic information necessary for keypoint correlation.

#### 3.3.2. Transformer Encoder

The encoder (6 layers) uses multi-head self-attention to model the relationships between all pairs of features in the sequence, enabling the capture of long-range dependencies (e.g., between marker corners and nearby structural edges). A critical design is the fused positional encoding, which combines the following:

1. Absolute positional encoding: Encodes pixel coordinates (x,y) in the ROI to preserve spatial layout.
2. Geometric prior encoding: Encodes marker-specific priors (center coordinates: $\left({\mathrm{center}}_{x},{\mathrm{center}}_{y}\right)$, width: *w*, height: *h*) derived from Stage 1’s coarse bounding box, anchoring attention to the marker geometry.

The positional encoding equation can be written as

(6) $$X_{\mathrm{pos}}=X+\mathrm{PE}\left({\mathrm{center}}_{x},{\mathrm{center}}_{y},w,h\right)$$

where PE(·) encodes the marker center coordinates and aspect ratio. The multi-head attention operation is

(7) $$\mathrm{MultiHead}\left(Q,K,V\right)=\mathrm{Concat}\left({\mathrm{head}}_{1},\ldots ,{\mathrm{head}}_{h}\right)W^{O}.$$

This splits features into subspaces to model diverse correlations (e.g., local shape vs. global context).

#### 3.3.3. Homography Decoding

After encoding, global average pooling (GAP) aggregates the sequence into a compact feature vector $\hat{X}\in R^{C}$, capturing the global scene context. A 3-layer multi-layer perceptron (MLP) maps $\hat{X}$ to a 9-dimensional vector (flattened 3×3 matrix), normalized by the Frobenius norm to ensure scale invariance:

(8) $$H=\frac{\mathrm{MLP}(GAP\left(X_{enc}\right))}{{∥H∥}_{F}}$$

where ${∥H∥}_{F}=\sqrt{\sum_{i,j}H_{i,j}^{2}}$ prevents scale ambiguity in homography regression.

### 3.4. Multi-Dimensional Data Augmentation

To address the challenge of limited annotated data for object detection tasks, we propose a synthetic sample generation pipeline that combines geometric transformations, photometric augmentations, and background integration. This approach generates diverse training samples with corresponding YOLO-formatted bounding box annotations, simulating real-world variations in object appearance and context.

#### 3.4.1. Random Homography Transformation

First, we apply a small-magnitude random homography transformation to the target template to simulate viewpoint variations (e.g., rotation, scaling, shearing, and translation). The transformation matrix $H\in R^{3\times 3}$ is constructed with perturbations bounded by a parameter max_perturb (default: 0.1), which controls the maximum relative deviation from the identity matrix. Specifically,

- Scaling factors for *x*- and *y*-axes are perturbed by ±max_perturb;
- Shearing terms $H_{0,1}$ and $H_{1,0}$ are perturbed by ±max_perturb/2;
- Translation offsets $H_{0,2}$ and $H_{1,2}$ are limited to ±max_perturb×template_width and ±max_perturb×template_width, respectively.

After transformation, the output size is dynamically adjusted to avoid cropping by aligning the minimum transformed coordinates to the top-left corner of the canvas.

#### 3.4.2. Photometric Augmentation

To enhance the sample diversity, we apply photometric transformations to the transformed template. This includes the following:

- Color space adjustments: Random brightness (color_range∈[0.7,1.3]) and saturation modifications in the HSV space, followed by contrast (α∈[0.7,1.3]) and brightness offset (β∈[−20,20]) adjustments in the BGR space;
- Gaussian noise injection: Additive Gaussian noise with variance noise_var∈[5,20] to simulate sensor noise or low-light conditions.

#### 3.4.3. Background Integration and Bounding Box Extraction

**Figure 5:**
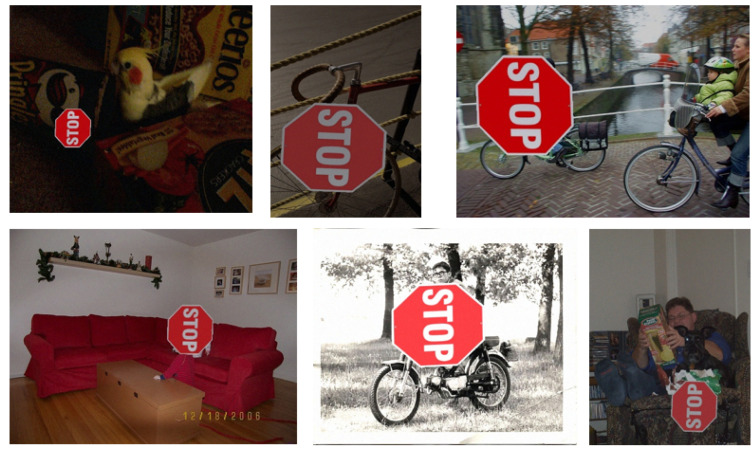
Examples of generated image samples.

The augmented template is then randomly pasted onto a background image (selected from a predefined dataset: VOC2007) while ensuring full containment within the background dimensions. The VOC2007 dataset is part of the PASCAL VOC project; it encompasses 9963 images and covers diverse scenes like urban streets, indoor rooms, and natural landscapes. A binary mask (thresholded at 1 pixel intensity) is used to identify the non-black (foreground) region of the transformed template, from which the minimum bounding box $\left(x_{1},y_{1},x_{2},y_{2}\right)$ is extracted. This bounding box is translated to absolute coordinates relative to the background image. Several examples of generated image samples are shown in Figure 5.

### 3.5. Loss Function

The first-stage detection loss follows the YOLOv11 formulation, which includes localization, confidence, and classification components, as defined in the original YOLOv11 architecture.

The second-stage loss for homography matrix $H\in R^{3\times 3}$ uses Smooth L1 to regress the predicted $\hat{H}$ to the ground-truth $H^{*}$:

$$L_{\mathrm{homo}}=\frac{1}{9}\sum_{k=1}^{9}\mathrm{SmoothL}1\left({\hat{H}}_{k}-H_{k}^{*}\right)$$

## 4. Experimental Setup and Result Analysis

### 4.1. Experimental Setup

The proposed network is trained on a PC with an NVIDIA GeForce RTX 2080Ti GPU (Santa Clara, CA, USA) and 64 GB RAM. The network is implemented with PyTorch 1.10.0 with CUDA 11.3. Training employs a learning rate of 0.001 and batch size of 16 for 100 epochs, with the Adam optimizer and cosine annealing for learning rate scheduling.

### 4.2. Experimental Datasets and Preprocessing

The Third Construction Co., Ltd. of China Construction Eighth Engineering Bureau, Nanjing, China.

#### 4.2.1. Real Construction Dataset

The dataset was collected from 3 bridge construction sites (The Third Construction Co., Ltd. of China Construction Eighth Engineering Bureau, Nanjing, China) over 2 months (April–May 2024) using a Intel RealSense Depth Camera D455 (Santa Clara, CA, USA) (12 MP resolution, 30 fps) mounted on the bridge-erecting machine’s hoist arm. The dataset includes 2000 images with the following:

- Markers: 5 types of rectangular markers (10–20cm in size) affixed to precast girders;
- Annotations: Bounding boxes (labeled via LabelImg v1.8.5) and 4 corner coordinates (manually verified for sub-pixel accuracy using OpenCV4.0’s cornerSubPix);
- Disturbances: 32% with lighting variations (morning/afternoon sun, overcast), 28% with partial occlusion (crane arms, worker bodies), 21% with contamination (dust, paint peeling)—a distribution aligned with field observations.

#### 4.2.2. Augmented Dataset

Following the multi-dimensional augmentation strategy described in Section 3.4, 5000 synthetic images were generated by simulating interference factors (lighting distortion, random occlusion, marker contamination). These synthetic data were mixed with real data at a 1:1 ratio for training.

### 4.3. Evaluation Metrics

For the detection performance evaluation, the mean average precision (mAP) with an intersection over union threshold of 0.5 was used to evaluate the marker detection accuracy.

For the localization precision evaluation, the homography reprojection error is adopted, which is the average pixel difference between the predicted and ground-truth coordinates of marker corners after applying the estimated homography matrix.

For the real-time performance evaluation, the frames per second (FPS) was measured to evaluate the computational efficiency.

#### Robustness

Robustness was assessed by measuring the mAP and treprojection error on three challenging subsets:

- Images with lighting variations;
- Images with partial marker occlusion;
- Images with contaminated markers (e.g., dust adhesion, paint peeling).

### 4.4. Comparative Experiments

Three comparative methods were tested to validate the proposed network’s superiority:

1. **Single-stage detection + traditional homography**: YOLOv8 (single-stage detector) combined with SIFT feature matching for homography estimation;
2. **Two-stage CNN network**: Faster R-CNN (two-stage detector) followed by a CNN-based homography regression module;
3. **Proposed method without data augmentation**: Identical to the proposed network but trained using only real data (no augmented samples).

### 4.5. Ablation Studies

Three key components were ablated to verify their contributions:

1. **Transformer module**: Performance comparison between the proposed network and a variant where the Transformer-based homography estimation module was replaced with a CNN regression network.
2. **Data augmentation**: Comparison of mAP and reprojection error with/without using augmented training data.
3. **Positional encoding**: Evaluation of three variants:
  - Using only absolute positional encoding;
  - Using only geometric prior encoding (marker center coordinates and aspect ratio);
  - Using the proposed fused positional encoding (absolute + geometric).

### 4.6. Result Analysis

#### 4.6.1. Quantitative Results

Table 1 presents the performance metrics as mean values ± standard deviations from three independent training runs, confirming the result stability. The proposed method exhibits the lowest variability across all metrics (e.g., mAP std. dev. = 0.4%), indicating robust convergence and consistent performance.

During inference, the lightweight marker detection in Stage 1 has average memory usage of 4.2 GB and an inference time of 19 ms per image (1280 × 960). The homography estimation in Stage 2 has average memory usage of 5.8 GB and an inference time of 12 ms per image. The total end-to-end inference time is 31 ms per image.

#### 4.6.2. Ablation Study Results

**Transformer Module vs. CNN Regression.** Table 2 shows that replacing the Transformer-based homography module with a CNN regression network reduces the mAP by 2.8% and increases the reprojection error by 0.8 mm. This gap stems from CNNs’ focus on local correlations; thus, they fail to model long-range dependencies between marker keypoints and distant scene structures—critical for handling occlusion or contamination in construction. The Transformer’s multi-head attention captures these global relationships, enabling robust estimation even when markers are partially obscured (e.g., by crane arms), whereas CNNs’ limited receptive fields lead to underfitting.

**Impact of Data Augmentation.** Without data augmentation, the mAP drops by 1.7% and the reprojection error rises by 0.6 mm, confirming that our multi-dimensional strategy (random homography, photometric changes, background blending) mitigates overfitting to limited real data. Synthetic samples simulate dust, variable lighting, and other disturbances, introducing diverse texture and illumination variations. This forces the model to learn invariant features, enhancing its generalization to the highly variable conditions of real construction sites.

**Positional Encoding Variants.** Fused positional encoding (absolute + geometric priors) outperforms single components, with a 1.3% higher mAP and 0.4 mm lower error than absolute encoding alone and a 1.5% higher mAP with a 0.5 mm lower error than geometric priors alone. Absolute encoding preserves fine-grained spatial details (e.g., marker edges), while geometric priors (center, dimensions from Stage 1) provide structural constraints. Their fusion balances local precision and the global context, preventing drift from noise or occlusion—vital for millimeter-level hoisting alignment.

Table 3 shows additional experiments on data ratios. The 2:5 ratio outperforms the others, confirming its rationality as it balances real-world representation and synthetic diversity. It is noted that the current disturbance definitions (e.g., marker contamination, occlusion) are simplified representations of real-world complexities. While we focused on dust and paint peeling for contamination and random shapes for occlusion, real scenarios may involve oil stains, rust, linear obstructions (e.g., mechanical arms), or diffuse obstructions (e.g., dust clouds). However, our data augmentation strategy prioritized capturing core disturbance features: texture degradation for contamination and spatial obstruction for occlusion. The proposed two-stage network, with its dynamic feature adaptation (via DHB) and long-range dependency modeling (via Transformer), learns generalized patterns beyond specific disturbance types. This is supported by its robust performance on the existing disturbed subsets, suggesting potential adaptability to unlisted disturbances. Future work will expand the disturbance library to cover more specific cases, further validating the model’s generalization ability.

#### 4.6.3. Scalability Analysis

To assess the performance under varying operational conditions, we generated test data by randomly stitching two to four original images (640 × 480) to create higher resolutions (1280 × 960, 1920 × 1440, 2880 × 2160), with 2, 4–5, and 6–8 markers per frame, respectively. The results are shown in Table 4. The model maintains high accuracy (mAP > 96%) and low errors (<1.6 mm) across the stitched conditions.

#### 4.6.4. Qualitative Analysis

**Figure 6:**
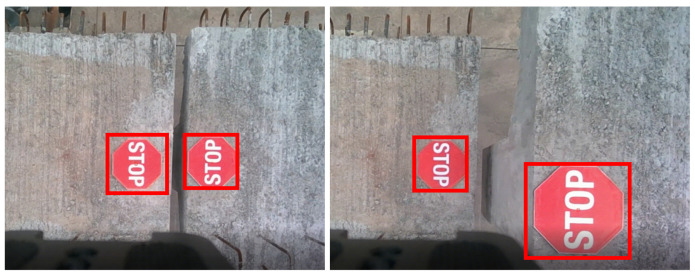
Examples of detection results.

**Figure 7:**
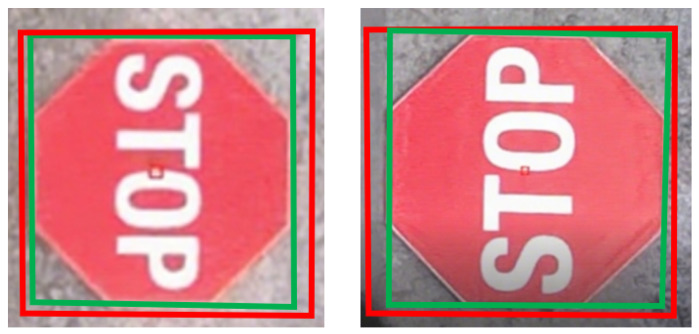
Examples of homography estimation results.

Figure 6 presents the partial marker detection results. The detector can effectively detect the targets, with no false positives or false negatives observed. Figure 7 shows the precise estimation results for homography (the green bounding box in the figure). We can see that the output results of the detector cannot accurately locate the boundaries of the markers. However, after our homography estimation, the obtained bounding boxes precisely align with the markers, thus meeting the requirement for high-precision localization.

#### 4.6.5. Robustness Verification

In extreme scenarios, the results are as follows:

- Occluded markers: mAP=95.6% (as shown in Table 1);
- Contaminated markers: reprojection error =1.5mm.

These results validate the network’s strong engineering applicability under complex construction conditions.

## 5. Conclusions and Discussion

The proposed two-stage network, which integrates lightweight detection with Transformer-driven homography estimation, successfully achieves high-precision and real-time marker detection and localization in complex construction environments. Additionally, the multi-dimensional data augmentation strategy effectively mitigates the challenge of limited real-world data availability, thereby enhancing the model’s generalization performance. Furthermore, the experimental results validate the robustness and practicality of the proposed method in engineering scenarios, providing critical technical support for automated hoisting operations by bridge-erecting machines.

To further advance this research, several key directions are identified. First, multi-modal fusion will be explored by incorporating laser point cloud or infrared sensor data to improve the alignment accuracy under low-light or dusty conditions. Second, model lightweighting will be pursued through techniques such as knowledge distillation or quantization compression, aiming to reduce the computational complexity and enable deployment on embedded devices (e.g., bridge-erecting machine controllers) while maintaining real-time performance. Third, dynamic scenario extension will be addressed by introducing temporal modeling (e.g., combining Transformer with LSTM) to handle dynamically moving markers during hoisting, thereby enhancing the stability of continuous frame alignment.

## Figures and Tables

**Table 1:** Comparison of Detection, Localization and Real-Time Performance (mean ± std. dev. over 3 runs).

Method	Detection	Localization	Real-Time	Robustness
	mAP (%)	Reprojection Error (mm)	FPS	${\mathrm{mAP}}_{\mathrm{occlusion}}$ (%)
YOLOv8+SIFT	90.2 ± 0.8	3.5 ± 0.3	45 ± 1	82.1 ± 1.2
Faster R-CNN+CNN	93.4 ± 0.6	2.3 ± 0.2	38 ± 1	88.7 ± 0.9
Ours (no augmentation)	96.1 ± 0.5	1.8 ± 0.2	32 ± 1	92.4 ± 0.7
**Proposed Method**	**97.8 ± 0.4**	**1.2 ± 0.1**	**32 ± 1**	**95.6 ± 0.5**

**Table 2:** Ablation study on key components.

Variant	mAP (%)	Reprojection Error (mm)	FPS
Full model (proposed)	97.8	1.2	32
Without Transformer (CNN regression)	95.0	2.0	33
Without data augmentation	96.1	1.8	32
Absolute pos. encoding only	96.5	1.6	32
Geometric prior encoding only	96.3	1.7	32

**Table 3:** Performance under different real-to-synthetic data ratios.

Ratio (Real–Synthetic)	mAP (%)	Reprojection Error (mm)	FPS
1:1 (2000:2000)	96.9	1.4	32
2:5 (2000:5000)	97.8	1.2	32
3:5 (3000:5000)	97.2	1.3	32

**Table 4:** Performance under stitched images (varying resolutions/marker counts).

Stitched Condition	mAP (%)	Reprojection Error (mm)	FPS
1280 × 960, 2 markers	97.3	1.3	30
1920 × 1440, 4–5 markers	96.5	1.5	12
2880 × 2160, 6–8 markers	96.1	1.6	4

## Data Availability

The original contributions presented in the study are included in the article; further inquiries can be directed to the corresponding author.
